# Managing Osteoporosis: A Survey of Knowledge, Attitudes and Practices among Primary Care Physicians in Israel

**DOI:** 10.1371/journal.pone.0160661

**Published:** 2016-08-05

**Authors:** Yacov Fogelman, Inbal Goldshtein, Elena Segal, Sofia Ish-Shalom

**Affiliations:** 1 Department of Family Practice, Leumit Health Services Tel aviv, Israel; 2 The Ruth & Bruce Rappaport Faculty of Medicine, Technion—Israel Institute of Technology, Haifa, Israel; 3 Epidemiology and Database Research, Maccabi Healthcare Services, Tel-Aviv, Israel; 4 Tel Aviv University, Tel Aviv, Israel; 5 Department of Endocrinology, Rambam Health Care Campus, Haifa, Israel; 6 Elisha Hospital, Haifa, Israel; Tel Aviv Sourasky Medical Center, ISRAEL

## Abstract

**Background:**

Osteoporosis is a systemic skeletal disorder characterized by impaired bone quality and microstructural deterioration leading to an increased propensity to fractures. This is a major health problem for older adults, which comprise an increasingly greater proportion of the general population. Due to a large number of patients and the insufficient availability of specialists in Israel and worldwide, osteoporosis is treated in large part by primary care physicians. We assessed the knowledge of primary care physicians on the diagnosis and treatment of osteoporosis.

**Methods:**

Physician's knowledge, sources of knowledge acquisition and self-evaluation of knowledge were assessed using a multiple choice questionnaire. Professional and demographic characteristics were assessed as well.

**Results:**

Of 490 physicians attending a conference, 363 filled the questionnaires (74% response rate). The physicians demonstrated better expertise in diagnosis than in medications (mechanism of action, side effects or contra-indications) but less than for other treatment related decisions. Overall, 50% demonstrated adequate knowledge of calcium and vitamin D supplementation, 51% were aware of the main therapeutic purpose of osteoporosis pharmacotherapy and 3% were aware that bisphosphonates should be avoided in patients with impaired renal function. Respondents stated frontal lectures at meetings as their main source of information on the subject.

**Conclusion:**

The study indicates the need to intensify efforts to improve the knowledge of primary care physicians regarding osteoporosis, in general; and osteoporosis pharmacotherapy, in particular.

## Introduction

Osteoporosis is a systemic skeletal disorder characterized by impaired bone quality and microstructural deterioration, leading to an increased propensity to fractures. This is a major health problem for older adults, who comprise an increasingly greater proportion of the general population. Over 10 million adults in the United States are estimated to have osteoporosis and an additional 43 million to have low bone mass [1]. Osteoporosis poses a serious worldwide health economics issue, though secular and temporal trends differ considerably by region [2]. Medical, social and economic consequences of the disease include fractures resulting in disability [3–5], excess mortality [6] and rising costs [7]. Recent publications document inadequacies in diagnosis, prevention and treatment following osteoporosis-related fractures [8–11].

An osteoporosis registry was recently established in Israel, by Maccabi Health Services [12], an HMO that insures 25% of the Israeli population. The identification of 118,141 osteoporosis patients suggests that the total number of patients in Israel may be nearing 500,000. Israel’s national health insurance law (enacted since 1995) provides every citizen with a universal compulsory health coverage financed by general taxation. This includes cradle to grave membership in any of the 4 competing HMOs, as well as adequate and affordable access to a government defined package of health services, including diagnostic tools and reimbursement of pharmacotherapy. Due to a large number of patients and an insufficient availability of specialists (endocrinologists and rheumatologists) in Israel, osteoporosis is treated in large part by primary care physicians. In other healthcare systems throughout the world, general practitioners and family medicine specialists also have central roles in identifying patients at risk for osteoporosis, and in diagnosing and managing the disease [13–16]. Thus, updated and adequate knowledge of osteoporosis by primary care physicians is of high importance. We distributed a questionnaire to assess the knowledge in this population of the diagnosis of osteoporosis and of the mechanisms, indications and side effects of currently available medications for osteoporosis.

## Materials and Methods

### Study procedure

This is a cross-sectional study of primary care physicians who filled a questionnaire. The questionnaire (see Supporting Information) was based on guidelines of the 2012 Israeli Foundation for Osteoporosis and Bone Diseases (IFOB). Multiple choice questions were used to assess information pertaining to the diagnosis and treatment of osteoporosis: knowledge of the various medications, including their mechanisms of action and side effects, as well as knowledge of the recommended dosage of vitamin D supplementation. The physicians were also asked about their sources of knowledge acquisition and how they keep updated on this topic, as well as their self-evaluation of knowledge. Additionally, background information was collected including professional and demographic characteristics. The content validity of the questionnaire was established by 2 experts in osteoporosis. Thirty-two physicians reviewed the questionnaire in face-to-face- interviews.

The Institutional Review Board of Rambam Health Care Campus exempted the study from ethical approval since it involved physicians only. Nonetheless, participants provided their written informed consent to participate in this study. The hospital ethics committee approved this consent procedure.

### The study population

The questionnaires were filled by physicians who participated in weekly continuing medical education meetings between January and March 2014, at 14 centers across the country. The centers were randomly selected from 89 centers that provide continuing medical education for 75% of primary care physicians in Israel. Participants at the meetings were general practitioners, and specialists and residents in family medicine at various levels of seniority and training. The questionnaires were filled anonymously and without compensation.

### Statistical methods

The pattern of item- non- response (missing answers) was examined to distinguish between missing at random (MAR) and missing not at random, and to identify potential evidence for fatigue or lack of cooperation (for example a participant who skipped all questions from a certain point in the questionnaire). No such evidence was observed. In addition, due to the nature of the survey; i.e. short and administered in a closed environment, we assumed that item non response indicated that participants were uncertain or did not know the answer. Missing answers were thus considered as incorrect. For questions with more than 1 correct statement (1–4, 13) an overall correct response was defined as marking all correct statements and not marking any of the incorrect statements

Thus, for each question analyzed, we calculated the proportion of respondents that answered correctly out of all physicians who filled the questionnaire (n = 363 survey participants).

Age and seniority of study participants were described by means and standard deviations, and compared across professional status (general practitioner, family medicine specialist and family medicine resident) using ANOVA. The total test-score per respondent was calculated as the number of correct answers, including all possible choices of all questions, divided by 38 (the overall number of questions including all possible answers), so that the range of scores is 0–100. Test scores were compared across levels of perceived knowledge, age (<40, 40–59 and 50+ years old), seniority (<10, 10–24 and 25+ years), gender and professional status, using ANOVA.

Responses regarding the recency of participation in lectures on osteoporosis were presented by the percentage of this question’s total number of respondents (i.e. excluding 22% item non-respondents).

Physicians were asked to rate sources of knowledge from 1–5; the higher the score, the greater the significance. We regarded rates of 4–5 as significant resources, and summarized the percentage of participants that rated each resource as significant (n = 363). The same approach was used for analysis of constraints to optimal management.

## Results

Of the 490 physicians that we approached at 14 centers of continuing medical education meetings, 363 (74%) filled and returned the questionnaires.

### Physician characteristics

Of the 363 physicians who completed the questionnaires, 86 (52%) were female. Mean ages and seniority, according to professional status, are presented in Table 1. Age was available for 349 participants, seniority was available for 338 participants, gender was available for 356 participants and professional status was available for 359 participants.

**Table 1:** Professional status, age and seniority (number of years in practice) of participating physicians.

Characteristics	All	Professional status		
		Residents	Board certified family specialists	General practitioners
No. of physicians	363 (100%)^1^	66 (18%)	155 (43%)	138 (38%)
Age (mean ±SD) (P<0.001)	46.0±9.9	32.4±5.0	46.4±7.5	52.1+7.4
Seniority (years)(mean ±SD) (p<0.001)	17.8±9.8	4.3±4.1	18.0±7.3	24.5±7.3

Table 2 summarizes success rates of the participants, per question (see detailed findings in the next 4 sections). The physicians demonstrated better expertise in diagnosis than in medications (mechanism of action, side effects or contra-indications) but less than for other treatment related decisions.

**Table 2:** Summary of survey results: knowledge of primary care physicians on the diagnosis and treatment of osteoporosis (n = 363).

	Item	Correct responses, n	Item non response, n (%)	% of correct responses after excluding item non-response	% of correct responses out of total survey participants
Diagnosis	Diagnostic tests	30	16 (4%)	9%	8%
	Treatment initiation	68	15 (4%)	20%	19%
	Fracture correlates	68	4 (1%)	19%	19%
	Clinical fracture risk factors	28	5 (1%)	8%	8%
Prevention	Vitamin D & Calcium	180	16 (4%)	52%	50%
Treatment decision	Treatment goal, 66 y/o	185	17 (5%)	54%	51%
	Recommendation, 54 y/o	164	11 (3%)	47%	45%
	Treatment duration	186	12 (3%)	53%	51%
Medications	Mechanism of action	7	119 (33%)	3%	2%
	DXA in Osteoporosis	82	86 (24%)	30%	23%
	Atypical fracture	83	59 (16%)	27%	23%
	Side effects	0	276 (76%)	0%	0%
	Contraindications	10	37 (10%)	3%	3%

The table presents the numbers of surveyed participants who answered each item correctly and the numbers and percentages who left each item blank. The percentage of correct responses is calculated first after excluding the blank answers from the total; and then without excluding the blank answers (the blank answers are considered incorrect). Non-response for at least one of the question subsections was considered an item non-response.

### Osteoporosis diagnosis

Four questions examined physicians’ proficiency in diagnosing osteoporosis. The first question was: "Which tests should be performed on a 60 year-old asymptomatic woman before determining the need for medical treatment of osteoporosis?" Only 8% of the physicians selected all three of the correct answers: 1) blood tests: levels of calcium, phosphorus, albumin, creatinine, blood count; 2) bone density scan using DXA; and 3) medical history and physical examination; and no wrong answers. However, 55% selected two of the three correct answers. The correct answer that was least selected was "blood tests", selected by 52%.

To the question: "Which patients should begin treatment, without further examination, to confirm diagnosis of osteoporosis?" only 19% selected both correct answers. However, 52% selected the correct response: "a 76 year-old man with an intertrochanteric hip fracture caused by falling from standing height after tripping on the carpet in his home"; and 44% selected the correct response "a 74 year-old woman with a sub-capital hip fracture caused by a fall in the garden while weeding".

The third question on diagnosis was: "What are the clinical factors associated with increased risk for osteoporotic fractures?" Only 19% selected all 5 of the correct answers (over the age of 65 years, female, current smoking, parental history of hip fractures, and current alcohol consumption of more than 3 servings per day). The risks of older age and female sex were known by 73% and 57% of the respondents respectively. Only 65% and 63% knew that current smoking and current alcohol consumption, respectively, are also risk factors.

The fourth question on diagnosis was: "What are the medical conditions that increase the risk of osteoporotic fractures?" Only 8% selected all 6 of the correct answers, and no wrong answers (chronic oral treatment with glucocorticoids, rheumatoid arthritis, type 2 diabetes, type 1 diabetes, hyperactive thyroid gland and primary hyperparathyroidism). However, 28% of the survey participants selected 5 of 6 of the correct responses. Most respondents (86%) knew that chronic oral glucocorticoid treatment, taken for more than 3 months, is a risk factor. The proportions that answered correctly regarding the other risk factors were 51%, 50%, 46%, 50% and 52%, respectively.

### Prescribing calcium and vitamin D supplements

One question assessed knowledge on this subject: "What is the recommended dosage of calcium and vitamin D for postmenopausal women?" A total of 50% of survey participants answered correctly that the dosage should be determined by a patient's dietary habits and lifestyle.

### Osteoporosis treatment decisions

Three questions assessed physicians’ knowledge on this subject. Of these questions, the highest proportion (51%) answered correctly that "reducing the risk of fracture by 25% to 50% in the various skeletal sites" is the therapeutic goal for a 66 year-old woman diagnosed with osteoporosis and treated with alendronate and calcium 600 mg/day. A total of 45% answered correctly, that treatment should remain unchanged for a 54 year-old woman with severe menopausal symptoms whose quality of life has greatly improved during one year of hormonal treatment (combined 1 mg estradiol/ 0.5 mg norethisterone acetate; her bone mineral density (BMD) T-SCORE was—3 for lumbar vertebra and -2.4 for femoral neck). The third question was: "What is the maximum treatment duration with various bisphosphonates for which fracture risk reduction efficacy was demonstrated in postmenopausal women?" A total of 51% selected the correct answer, 3–6 years.

### Pharmacotherapy for osteoporosis

Physicians were asked to classify medications for osteoporosis by their mechanisms of action: anti-resorbing or anabolic. The proportions of physicians that answered correctly were 52%, 42%, 39%, 47%, 17% and 32% for alendronate, raloxifene, teriparatide, residronate, denosumab and zoledronate, respectively.

Twenty-nine physicians (8%) did not answer the question pertaining to side effects of osteoporosis medications completely, while n = 276 (76%) skipped at least one of its subsections. Only 9 physicians (2%) correctly matched at least one side effect for each of the medications listed above. The proportions that answered correctly per medication were: 56% for alendronate, 47% for raloxifene, 8% for teriparatide, 49% for risedronate, 5% for denosumab and 9% for zoledronate. Only 3% of the physicians correctly selected alendronate, residronate and zoledronate as the three medications that should not be administered to a patient with eGFR <35.

A total of 23% of the physicians participating in the survey provided the correct answer ("none of the above") to a question pertaining to the follow-up of a patient with osteoporosis using a BMD test with DXA.

The question "What characterizes an atypical hip fracture?" was answered correctly by 23% of the physicians: as a femoral shaft fracture in the subtrochanteric site, associated with prolonged use of bisphosphonates.

### Differences in knowledge about osteoporosis according to physician characteristics

Physicians aged <40 years demonstrated better overall knowledge than physicians aged 40–59 or aged 50+ years: mean total scores were 48, 46 and 44, respectively (p = 0.006). A high correlation was observed between age and years of practice, and thus the trend of total scores according to seniority was similar to that of age: mean total scores were 48, 47 and 44 for physicians with <10, 10–24 and 25+ seniority years, respectively (p = 0.035). No statistically significant differences were found in overall knowledge between male and female physicians (p = 0.63). Only 5% perceived their knowledge as high, 41% rated it as adequate, 46% as low and 8% did not respond to this question. No association was found between perceived knowledge and knowledge measured by the questionnaire (p = 0.42). Board certified specialists (mean total score 48) and residents (mean total score 49) in family medicine demonstrated better overall knowledge of osteoporosis than did general practitioners (mean total score 42, p<0.001) but similar knowledge of pharmacology, as demonstrated by responses regarding the mechanism of action of medications such as teriparatide (p = 0.79) and denosumab (p = 0.46).

### Sources of Knowledge about Osteoporosis

Physicians most often answered that expert lectures in continuing medical education and professional conferences, (59%) and clinical experience (58%), were significant sources of knowledge pertaining to osteoporosis. A total of 31% responded that they had attended a lecture on the subject within the past two years (Table 3).

**Table 3:** The proximity of the date of physicians' participation in lectures on osteoporosis.

Time from last lecture on osteoporosis	n (% of responders)
0–3 months	2 (1%)
3–6 months	4 (1%)
6–12 months	15 (5%)
12–24 months	68 (24%)
2–5 years	107 (38%)
Over 5 years	89 (31%)
Did not answer	78

### Constraints to optimal management

Physicians answered that "lack of consistent compliance by patients" (26%) and "inadequate knowledge in the field" (24%) were the major constraints to provision of optimal management for osteoporosis. Other constraints reported were: bureaucratic problems (filling forms for approval of osteoporosis medications) (18%), expenses of medications (17%), time limitation of the medical consultation (20%), the lack of evidence-based diagnostic tools (11%), side effects of medications (7%) and lack of faith in the efficacy of available medications (4%).

## Discussion

Studies conducted around the world have shown inadequate knowledge about osteoporosis among family physicians [17–24]. Most studies have investigated knowledge about risk factors and the conditions that should prompt evaluation. Little research attention has been given to osteoporosis medications, despite the availability in recent years of a multitude of drugs and new treatments. In the present study, pharmacotherapy was the topic with which physicians had the most difficulty. Large gaps in knowledge were revealed regarding mechanisms, side effects and indications of available medications. As expected, physicians exhibited more knowledge of the mechanisms of action and the side effects of the osteoporosis medications that are more frequently administered at primary care clinics: namely drugs from the bisphosphonate group.

Forty-six percent of the surveyed physicians stated that their knowledge of osteoporosis was adequate or high. This compares with the findings of a German study in which 50% of the respondents reported familiarity with the most recent guidelines on the subject [20].

Family medicine specialists displayed greater knowledge of osteoporosis diagnosis and treatment than did general practitioners, concurring with other studies [18]. Physician sex was not associated with level of knowledge about osteoporosis. This contrasts with other studies that reported women to be more knowledgeable [20–22], an observation explained by an overall greater awareness of women of the topic. Seniority and age of physicians in the current study were inversely associated with level of knowledge about osteoporosis. This concurs with studies that reported greater knowledge about osteoporosis among younger physicians [14,18, 21] and those with less seniority [18]. Minor differences in the present study may be due to the participation of physicians of all ages in weekly training courses provided by Israel’s HMOs.

The main constraints to optimal treatment for osteoporosis, according to the physicians who participated in the current study, are lack of consistent compliance on the part of patients and lack of knowledge on the part of physicians. Recently published studies have shown suboptimal adherence to osteoporosis treatment in a number of countries [11,25]. In other studies physicians stated costs and authorizations of diagnosis and treatment as the main limiting factor to optimal treatment [14]. The relatively low proportion of physicians that mentioned costs as the main limiting factor in the current study probably reflects the accessibility of diagnostic tests and the inclusion of diagnostic and treatment costs in the expenditures of the national healthcare system in Israel [12].

The topic about which the respondents exhibited the greatest knowledge was treatment decisions. However, the questionnaire was only able to evaluate knowledge and attitudes, and not actions. A number of studies have demonstrated knowledge to be only one of the barriers to the provision of osteoporotic treatment by primary care physicians. For example, though most general practitioners responding to a questionnaire recognized the importance of investigating osteoporosis among individuals over age 50 years with low trauma fractures, the majority stated they would only initiate such evaluation if prompted by an orthopedic surgeon [19]. In another study, only 31% of general practitioners initiated osteoporosis treatment for postmenopausal women admitted to the emergency department for peripheral fractures, despite dissemination of information on the matter to the physicians [13]. Such studies highlight the multidisciplinary nature of osteoporosis diagnosis and management. Recent publications have attributed deficiencies in osteoporosis management to inadequate communication and cooperation among the physicians involved: general practitioners, orthopedic surgeons, endocrinologists and rheumatologists [26,27].

The 74% response rate is a strength of the current study. The high participation rate may be due to the method of administration of the questionnaires, frontally with a time limit of 15–20 minutes, rather than online completion as in other studies. The questionnaires were filled on a voluntary basis, with no financial incentive.

A limitation of this study is the uncertainty of its generalizability. The findings presented herein reflect the knowledge of Israeli physicians who participated in the weekly in-service study seminars organized by the departments of family medicine and the HMOs in Israel. In principle, participation in these seminars is mandatory, but in practice not all primary care physicians attend them. Physicians who attend these seminars may be those who consider improving their professional knowledge as important. Missing data is a limitation of the study, and was particularly evident regarding side effects of medications.

The study indicates the need to intensify efforts to improve the knowledge of primary care physicians regarding osteoporosis, in general; and osteoporosis pharmacotherapy, in particular. This is especially important due to the central role of primary care physicians in Israel, as well as in many other regions, in the diagnosis and management of osteoporosis.

## Supporting Information

S1 Fig Osteoporosis Questionnaire. (DOCX)

S2 Fig Osteoporosis Questionnaire Answers. (XLSX)
