# Supplementary material for: Managing Osteoporosis: A Survey of Knowledge, Attitudes and Practices among Primary Care Physicians in Israel
Source: PLoS One. 2016 Aug 5;11(8):e0160661. doi: 10.1371/journal.pone.0160661 (PMC4975485; doi:10.1371/journal.pone.0160661)
Supplement: S1 Fig — (DOCX) [file pone.0160661.s001.docx]

**Approach to Diagnosis and Treatment of Osteoporosis among Israeli Family Physicians – A Questionnaire**

The World Health Organization lists Osteoporosis among the ten most significant diseases. Osteoporosis causes a decline in quality of life, greater morbidity, and a rise in mortality and high financial costs to the health system, mainly in the treatment of osteoporotic fractures. This questionnaire is designed to assess your approach to the diagnosis and treatment of osteoporosis.

1. What are the tests you would perform for a 60-year-old asymptomatic female patient before deciding on the need for osteoporotic pharmacotherapy? (You may choose more than one answer.)

1. Blood tests: levels of calcium, phosphorus, albumin, creatinine, blood count.
2. Bone density scan using DXA
3. Ultrasound bone density scan, the device is available for use at a nearby private clinic
4. X-ray of thoracic and lumbar vertebrae
5. Medical history and physical examination
6. Bone scan

2. For which of the following patients would you start treatment **without further testing for confirming diagnosis of osteoporosis**? (You may choose more than one answer.)

1. A 45 year-old female patient, generally healthy, regular menstrual cycle with TSCORE -2.9 in the spinal vertebra.
2. A 76 year-old male with an inter-trochanteric fracture in the hip caused by falling from a standing height after tripping on the carpet in his home .
3. A 68 year-old female, generally healthy, with a collapsed L4-vertebra after falling from a meter-high rock while hiking.
4. A 74 year-old female with a sub-capital hip fracture caused by a fall in the garden while weeding

3. What are the clinical factors associated with increased risk of osteoporotic fractures? (You may choose more than one answer.)

1. Over the age of 65
2. Female
3. Presently smoking
4. A parental history of femoral fractures
5. Present alcohol consumption of more than three servings per day

4. What are the clinical conditions that increase the risk for osteoporotic fractures? (You may choose more than one answer) –

1. Chronic oral glucocorticoid treatment (taken for more than 3 consecutive months)
2. Rheumatoid arthritis
3. Type 2 diabetes
4. Under-active thyroid gland
5. Type 1 diabetes
6. Overactive thyroid gland
7. Glucocorticoid treatment at a dosage of 40 mg with gradual reduction over the course of a week due to urticaria
8. Ischemic heart disease
9. Primary hyperparathyroidism

5. What is the recommended dosage of calcium and vitamin D supplement for postmenopausal women? (ONE correct answer only)

1. 200 units of vitamin D, 600mg calcium
2. 400 units of vitamin D, 1200mg calcium
3. 600 units of vitamin D, 1000mg calcium
4. 800 units of vitamin D, 600 mg calcium
5. Dosage to be determined by the patient’s dietary habits and lifestyle

6. What is the **therapeutic goal** for a 66 year-old patient diagnosed with osteoporosis and treated with FOSAVANCE and calcium 600 mg/day?

1. Increasing the bone density in spinal vertebra by at least 2% in two years
2. Reducing the risk of fracture by 25% to 50% in the various skeletal sites
3. Increasing bone density by 5% or more in the femoral shaft, within two years.
4. Increasing bone density by 6% within 5 years, in spinal vertebra.

7. A 54 year-old female with severe menopausal symptoms, has been taking hormone treatment (ACTIVELLE) for one year, which has greatly improved her quality of life. The patient took a bone density test as part of her periodic check-ups: T-SCORE for spinal vertebra:- 3 SD, and hip bone: -2.4 SD. She does not take additional medication, except for calcium 600 mg/day, and 1000 IU of vitamin D per day. Laboratory evaluation is within normal range. What is your recommendation? (ONE correct answer only)

1. Add FOSALAN 70 mg /week to ACTIVELLE
2. Stop ACTIVELLE treatment and begin treatment with EVISTA (RALOXIFEN)
3. Continue treatment unchanged
4. Add treatment with ACTONEL (RISEDRONATE) 150 mg/month to the ACTIVELLE treatment
5. Add treatment with ACLASTA (ZOLEDRONATE) by intravenous administration once a year
6. Stop ACTIVELLE treatment and begin treatment with FOSALAN 70 mg/week

8. The maximum treatment duration with various bisphosphonates for which fracture risk reduction efficacy was demonstrated in postmenopausal women is:

1. Two years
2. Three to six years
3. Unlimited duration of treatment
4. The period of time required for an increase in bone density of at least 6% in spinal vertebra
5. Duration can range from two to ten years, depending on the type of bisphosphonates administered.

9. Below is a list of medications used to treat osteoporosis in Israel. These drugs have different mechanisms of action.
If the drug is an anti-resorbing agent, mark a **1** beside it. If the drug is an anabolic agent, mark **2** beside it.

1. ALENDRONATE ( FOSALAN)
2. EVISTA ( RALOXIFEN)
3. FORTEO (TERIPARATIDE)
4. ACTONEL (RISEDRONATE)
5. PROLIA (DENOSUMAB)
6. ACLASTA (ZOLEDRONATE)

10. What is true about the **follow-up** for a patient with osteoporosis, using a bone mineral density test with DXA (ONE correct answer only)

1. The test is the most effective means for evaluating the response to pharmacotherapy at the individual level
2. This test is recommended annually for patients with severe osteoporosis, such as patients with a collapsed lumbar vertebra who are taking medication
3. This test is recommended once every two years for osteoporosis patients during treatment, to assess response to the treatment and recovery from the disease
4. If a decrease in bone density by 2% or more is observed, the medication needs to be adjusted
5. None of the above

11. What characterizes an ATYPICAL FRACTURE of the hip? (ONE correct answer only)

1. An osteoporotic fracture that occurs in the femur under the trochanter
2. A fracture that appears in a sub-trochanteric site in the FEMORAL SHAFT while a patient is being treated with TERIPARATIDE (FORTEO)
3. A fracture in the FEMORAL SHAFT in a sub-trochanteric site associated with prolonged use of bisphosphonates
4. A hard-to-heal osteoporotic fracture

12. The following is a list of medications used in treating osteoporosis and beneath it is a list of possible complications associated with the treatment. Please match the common complication to the medication that may trigger it (more than one complication per medication is possible).

1. FOSALAN(ALENDRONATE)
2. EVISTA ( RALOXIFEN)
3. FORTEO (TERIPARATIDE)
4. ACTONEL (RISEDRONATE)
5. PROLIA (DENOSUMAB)
6. ACLASTA (ZOLEDRONATE)
7. Venous thromboembolism event
8. Hypocalcemia
9. Musculoskeletal pain
10. Upper gastrointestinal bleeding
11. Epigastric pain and heartburn
12. Esophagitis
13. Erysipelas
14. Muscle pain, flu-like symptoms
15. Hypercalcemia
16. Aggravation of menopausal symptoms

13. Which of the following medications **should not** be administered to an osteoporosis patient with eGFR <35? (There may be more than one correct answer)

1. FOSALAN (ALENDRONATE)
2. EVISTA (RALOXIFEN)
3. FORTEO (TERIPARATIDE)
4. ACTONEL (RISEDRONATE)
5. PROLIA (DENOSUMAB)
6. ACLASTA (ZOLEDRONATE)

14. Rate your sources of knowledge regarding the approach to diagnosis and treatment of osteoporosis from 1-5, with 5 being the most significant source and 1 the least significant.

|  | 1 | 2 | 3 | 4 | 5 |
| --- | --- | --- | --- | --- | --- |
| A. Medical school |  |  |  |  |  |
| B. Clinical experience |  |  |  |  |  |
| C. Professional literature and self-education |  |  |  |  |  |
| D. Consultations with peers |  |  |  |  |  |
| E. Representatives of pharmaceutical companies at exhibitions and conferences |  |  |  |  |  |
| F. Expert lectures at continuing education seminars and conferences |  |  |  |  |  |
| G. The press, television |  |  |  |  |  |
| H. Other_____ |  |  |  |  |  |

15. When was the last time you attended an update lecture on osteoporosis:

1. 0-3 months ago
2. 3-6 months ago
3. 6-12 months ago
4. 12-24 months ago
5. 2-5 years ago
6. 5 years ago or more

16. You consider your level of knowledge on osteoporosis to be (circle the correct answer.):

1. High
2. Adequate for providing effective, quality care for patients
3. Low

17. In the table below, please rate the factors, from 1 to 5, that limit your ability to provide optimal care for your patients with osteoporosis, with 5 being the most limiting factor and 1 having the least influence.

|  | 1 | 2 | 3 | 4 | 5 |
| --- | --- | --- | --- | --- | --- |
| Lack of effective and fact-based means for assessing risk of fractures in patients |  |  |  |  |  |
| Lack of physician-patient time |  |  |  |  |  |
| Inadequate knowledge in the field |  |  |  |  |  |
| Bureaucratic difficulties |  |  |  |  |  |
| Side effects of presently administered medication |  |  |  |  |  |
| Lack of consistent compliance by patients |  |  |  |  |  |
| Patients’ financial hardships, which limit their ability to purchase medication |  |  |  |  |  |
| Lack of trust in the efficacy of the products for treatment of osteoporosis |  |  |  |  |  |

18. What in your opinion is the most effective way for you to update your knowledge about osteoporosis in the future?

1. Attending conferences for family physicians on the topic
2. Self-education from articles and books
3. Using a dedicated online tutorial for family physicians that grants study credits
4. Adding the topic to physicians' in-service seminars every 3, 6, 12 months (Circle the most appropriate frequency).

E. Discussion and presentations of cases to specialists in the field

Personal Details:
• Age: _____
• Gender: Male / Female
• Specialization:

- 1. Family Medicine intern
  2. Family Medicine specialist
  3. GP
  4. Other __________

• The number of years engaged in family medicine practice _____

Thank you very much for your participation. ☺
